# Prediction of Left Ventricular Reverse Remodelling: A Mini Review on Clinical Aspects

**DOI:** 10.1159/000526986

**Published:** 2022-09-14

**Authors:** Martin Chudý, Eva Goncalvesová

**Affiliations:** Department of Heart Failure and Transplantation, Faculty of Medicine, Comenius University and National Cardiovascular Institute, Bratislava, Slovakia

**Keywords:** Heart failure with improved ejection fraction, Reverse remodelling, Cardiac remission, Myocardial remission, Clinical predictors, Biomarkers

## Abstract

Improvement of left ventricular ejection fraction (LVEF) in patients after the first manifestation of heart failure with reduced ejection fraction (HFrEF) has currently been observed more frequently than it was years ago. This appears to be due to the early initiation of comprehensive HF therapy. According to these observations, a new HF syndrome category, heart failure with improved ejection fraction (HFimpEF), was introduced. In this short review, we present definitions of reverse remodelling, myocardial remission, and myocardial recovery. We provide an overview of clinical research aimed at evaluating reverse remodelling in different populations of patients with HFrEF. Clinical and imaging characteristics and biomarkers identified as predictors of reverse remodelling and improvement of the LVEF are discussed. We also briefly address the current views on the management of patients with HFimpEF. In-depth study and knowledge of the molecular mechanisms underlying the reverse remodelling process may lead to the identification of new individualized therapeutic approaches for HFrEF.

## Introduction

Myocardial injury, haemodynamic overload, and excessive neurohumoral activation are the main triggers of several pathophysiological processes leading to structural and functional changes in the myocardium, which are referred to as cardiac remodelling. This pathological process is responsible for the onset and progression of heart failure. Pathological remodelling causes hypertrophy, dilatation, and spherical left ventricular (LV) remodelling, as well as contractility disorders, and typically leads to heart failure with reduced ejection fraction (HFrEF). In the past, systolic dysfunction and remodelling were considered to be irreversible processes and were associated with an unfavourable prognosis. Currently, it is known that favourable changes in LV geometry and LV systolic function improvement can be observed in a significant percentage of patients with HFrEF. These morphological and functional myocardial changes are referred to as reverse remodelling (RR). RR is basically accompanied by termination or reversal of the pathophysiological processes responsible for the initial pathological remodelling. From a morphological point of view, the LV dimensions start returning to normal; mitral regurgitation becomes less severe, and the contractility and LV ejection fraction (LVEF) show improvement. In clinical practice, RR is associated with improved functional capacity and reduced hospitalization and mortality rates. According to these characteristics, the induction of RR becomes a major therapeutic target in patients with HFrEF. The predictive factors of RR have been partially described, but detailed knowledge of them, as well as accurate RR prediction, is important for a therapeutic strategy decision, especially when expensive device therapy is being considered.

## Reverse Remodelling

**Fig. 1:**
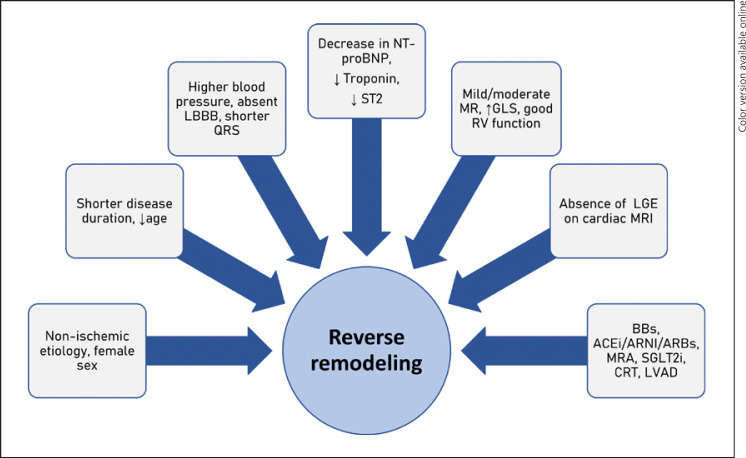
Factors associated with RR. ACEi, angiotensin-converting enzyme inhibitors; ARBs, angiotensin receptor blockers; ARNI, angiotensin receptor-neprilysin inhibitor; BBs, beta blockers; CRT, cardiac resynchronization therapy; LBBB, left bundle branch block; GLS, global longitudinal strain; LGE, late gadolinium enhancement; LVAD, left ventricle assist device; MR, mitral regurgitation; MRA, mineralocorticoid receptor antagonist; MRI, magnetic resonance; NT-proBNP, N-terminal prohormone of brain natriuretic peptide; RV, right ventricle; SGLT2i, sodium-glucose cotransporter-2 inhibitors; ST2, ST2 cardiac biomarker.

In recent years, there has been an increase in the number of studies focused on the group of patients with RR. This phenotype of heart failure is referred to as heart failure with improved ejection fraction (HFimpEF) or heart failure with recovered ejection fraction. Echocardiography plays a dominant role in the evaluation of RR. The presence or absence of RR is identified by the difference between the initial and follow-up values of the measured parameters (Fig. 1). The time interval between two measurements should be at least 3–6 months. The methodology and definition of RR criteria are considerably different in previous studies. Characteristics defining RR usually include a change in LVEF, a change in LV volumes, or a combination of both (Table 1). The reference ranges for LVEF and LV volume change are also not uniform. A recent study compared which of the mentioned parameters (EF change/LV volume change) characterize RR better, with the results showing a better correlation in the LVEF change. Despite the lack of guidelines and recommendations, there is consensus among experts on the following RR criteria [16]:

- documented reduced LVEF <40%,
- an increase in LVEF of at least 10% from the baseline value,
- an increase in LVEF to >40%.

Table 1 summarizes the basic characteristics and results of selected studies focused on LV RR in patients with HFrEF. The final incidence of RR induction ranges from 9.2% to 52%, which is also a result of the previously mentioned inconsistent study methodologies. The diversity of the studied populations also contributes to different RR incidences. The RR induction rate was higher in those studies focused on patients with idiopathic dilated cardiomyopathy (DCMP) and lower in populations of patients with heterogeneous causes of HFrEF. The average RR incidence is 22.64% according to a recent meta-analysis [17]. The same meta-analysis revealed a 56% lower death risk and a 60% lower hospitalization rate in patients with RR than in other HFrEF patients [17].

## Myocardial Remission and Myocardial Recovery

In the heart failure trajectory, we can observe various changes in clinical condition and LV systolic function. The stage of long-term disease stabilization, when patients are monitored on an outpatient basis, tolerate treatment well, and do not require hospitalization for decompensated heart failure, is referred to as *cardiac remission*. Cardiac remission may or may not be associated with improvement in LVEF, and therefore, it is clinically stabilized regardless of functional myocardial changes. If cardiac remission is associated with improvement in systolic function as well, we refer to this condition as *myocardial remission*. The term *remission* refers to a transient condition because despite the improved function, the cellular, molecular, and genetic processes are not in their physiological state, and these patients are still in danger of systolic dysfunction progression. In rare cases, complete normalization of pathological processes, including gene expression, may occur, which is typically accompanied by normalization of systolic function as well, which in practice means a cured patient. We also describe so-called *myocardial recovery* in these patients, but today, it is practically impossible to distinguish this stage from myocardial remission. Patients with myocardial remission represent the dominant portion of HFimpEF patients, as shown by several studies focused on this phenotype of patients. These studies confirm the persistence of abnormal gene expression as well as abnormal global longitudinal strain (GLS) of the left ventricle. After RR and improvement in LV systolic function due to LV assist device implantation, only approximately 5% of genes that are dysregulated in HF return to normal, despite morphological and functional heart recovery.

## Predictors of RR

### Aetiology and Clinical Parameters

The aetiology of heart failure is an important determinant of RR induction. In general, patients with an ischaemic cause of HFrEF are less likely to achieve RR than patients without ischaemic myocardial damage. Due to almost no ability of cardiomyocytes to regenerate, their postinfarction loss is practically irreversible, and if ventricular dysfunction is already present, no functional improvement is expected in the future. In contrast, due to the increased demands on the intact working myocardium, progression of the dysfunction is more likely to occur.

DCMP dominates among the nonischaemic causes of HFrEF, with nonfamilial forms of DCMP having a more favourable prognosis and a higher probability of RR induction. DCMP associated with truncating titin mutations usually responds well to drug treatment, and RR is more likely to develop than DCMP associated with laminin A/C and other sarcomere protein gene mutations. The estimated probability of RR induction due to proper treatment in patients with idiopathic DCMP is approximately 40% [2].

A relatively high rate of RR induction is also expected in patients with mild forms of myocarditis (e.g., lymphocyte myocarditis) [19], peripartum cardiomyopathy [20], and myocardial damage caused by various toxic agents. In these cases, the nature of the toxic substance, its dosage, and the duration of its action are very important. In patients with alcoholic myocardial damage, the degree of myocardial damage is dose-dependent, and in mild forms, improved function may be achieved by abstinence and appropriate drug treatment. In contrast, myocardial damage appears to be irreversible in patients after cardiotoxic cancer treatment (doxorubicin, daunorubicin, trastuzumab) [21]. Higher BMI [22], female sex [10, 13], shorter disease duration [11], and higher blood pressure [3, 12] were identified as predictors of RR derived from medical history and the patient´s clinical profile.

### Imaging

When using echocardiography in RR prediction, it is also necessary to evaluate, in addition to basic morphological and functional parameters, the deformation characteristics of the myocardium using speckle-tracking echocardiography as it reflects contractility and LV systolic function more reliably and seems to predict RR better. Some of the standard echocardiographic parameters are considered to be predictors of RR, including mild/moderate mitral regurgitation, higher TAPSE, smaller left atrial volume, and smaller LV end-diastolic volume. A larger end-diastolic dimension of LV and lower LVEF are paradoxical RR predictors, but these patients actually have a worse prognosis. However, an initially larger and more dysfunctional ventricle can more easily achieve an increase in LVEF even with a smaller change in LV geometry, but LVEF is unlikely to reach 40%; therefore, we should not consider these parameters as RR predictors. LV GLS assessment represents a more sensitive method for systolic function assessment that is relatively independent of ventricular volumes. Several studies have confirmed that GLS is a reliable RR predictor [23]. In a study on 160 patients with DCMP, RR was achieved in 28% of patients, and LV GLS proved to be its only independent predictor [15]. LV GLS also appears to be a suitable predictor of further clinical course in patients with HFimp­EF. Moreover, it has been shown that abnormal LV GLS in patients with HFimpEF predicts a further decrease in LVEF, while normal GLS predicts LVEF stabilization [24]. In addition to LV GLS assessment, left atrial GLS also seems to be a potential predictor. A recently published study on 100 patients with newly diagnosed HFrEF demonstrated left atrial GLS as a sensitive (96%) and specific (82%) RR predictor with a value >10.8% [25].

In addition to echocardiography, magnetic resonance imaging of the heart also plays an important role in RR prediction. Late gadolinium enhancement (LGE) represents the main parameter predicting RR. Patients with a low degree of LGE or no LGE at all are more likely to achieve RR [8, 26].

### Pharmacotherapy and Device Therapy

Today, the medical and device treatments used to improve the prognosis of HF patients also have a well-documented effect on reverse LV remodelling. Due to their long-term use, beta blockers and preparations that interfere with the renin-angiotensin aldosterone system (ACEi, ARB, MRA) have well-described mechanisms affecting RR. Newer drugs, including ARNI and SGLT2i, also have a demonstrated effect on RR, but no detailed explanation of their mechanism of action has been fully understood yet. Table 2 shows the effect of individual drugs on molecular, cellular, and extracellular changes responsible for RR.

### Biomarkers

Biomarkers are used routinely for heart failure diagnosis, prognosis assessment, and response to treatment monitoring. Currently, it is possible to evaluate the degree and severity of some pathophysiological processes accompanying the onset and progression of heart failure by using biomarkers [27]. The identification of biomarkers as RR predictors seems to be a useful step for clinical practice.

The contribution of NT-proBNP in RR prediction has been demonstrated in several studies that evaluated its dynamic changes. A significant NT-proBNP decrease was associated with LVEF improvement. In the Guiding Evidence-Based Therapy Using Biomarker Intensified Treatment (GUIDE-IT) study, which included 269 patients, a decrease in NT-proBNP levels below 1,000 ng/L at a time interval of 12 months after treatment initiation was correlated with LVEF improvement and LV volume reduction. No unambiguous correlation between the NT-pro­BNP initial value and RR prediction has been demonstrated in several larger studies.

Troponin T as a myocardial damage marker appears to be a promising RR predictor. A study on a heterogeneous group of HFrEF patients showed a higher incidence of RR in patients with an initial highly sensitive troponin T value <11 ng/L than in patients with higher troponin T levels [28].

Suppression of tumorigenicity 2 (ST2) is a marker of pathological remodelling that reflects the degree of fibrosis and LV hypertrophy in patients with HFrEF. Higher soluble ST2 levels have been associated with poorer prognosis of patients with HF in several studies [29, 30, 31]. One study including 304 outpatients with HFrEF identified ST2 as the only biomarker associated with RR in a multivariate analysis. The authors also proposed using the ST2-R2 score to predict RR, which consists of the following variables: ST2 < 48 ng/mL, nonischaemic aetiology, the absence of LBBB, disease duration <12 months, baseline LVEF <24%, and a history of beta blocker treatment (13). Other potential biomarkers of RR that are still being studied include mimecan [32], galectin-3 [33], IGF-binding protein-7 [32], and some micro-RNA subtypes and tissue inhibitors of metalloproteinases.

## Management of Patients with HFimpEF

After the definition of a new group of patients with heart failure, new questions regarding their management have arisen. Due to the incomplete understanding of various mechanisms of RR and a lack of large randomized clinical trials, no official guidelines for the management of patients with HFimpEF have yet been published, and only the consensus of experts is being followed.

A TRED-HF study addressed the issue of treatment termination or treatment continuation in patients with HFimpEF after LVEF and LV volume normalization and NT-proBNP decrease to less than 250 ng/L [34]. In that study, 51 patients with HmFrecEF due to DCMP were enrolled and randomized into two groups: a treatment-discontinued group (*n* = 25) and a treatment-continued group (*n* = 26), with an assignment ratio of 1:1. DCMP relapse (defined by a decrease in LVEF by >10%, an increase in LV volume by >10%, a doubling of NT-proBNP level with an increase to >400 ng/L, or development of clinical signs of heart failure) was considered the primary outcome. Within 6 months, 11 patients (44%) achieved the primary outcome in the discontinued-treatment group, while no patient achieved the primary outcome in the continued-treatment group. Subsequently, in the next 6 months, 25 patients from the continued-treatment group terminated their treatment, and 9 (36%) of them achieved a primary outcome within 6 months [34].

Repeated progression of the disease after previous recovery was examined in a study involving 85 patients with DCMP who continued with their heart failure therapy. Progression of systolic dysfunction after 50 ± 33 months was observed in 38% of cases and was associated with older age, greater LVEDD, and the presence of diabetes mellitus [35]. Discontinued diuretic therapy and the absence of signs of congestion are good indicators of disease stabilization in patients with HFimpEF.

Patients with HFimpEF should be monitored regularly on an outpatient basis for the risk of repeated progression of systolic dysfunction. The frequency of check-ups should be every 6 months during the first 3 years. The regular outpatient check-up should include clinical examination focused on signs of congestion, physical capacity evaluation, electrocardiogram, NT-proBNP level measurement, and echocardiography. In patients with cardiomyopathy with a higher risk of atrial arrhythmias, an ECG Holter of at least once every 2 years is recommended. The indication for primary prevention of sudden cardiac death in patients with HFimpEF is ambiguous, but ICD implantation should be considered in patients with an identified genetic cause of cardiomyopathy at high risk of ventricular arrhythmias (e.g., LMNA, SCN5A, FLNC).

It is already known that some patients diagnosed with HFpEF are actually patients who have already had a reduced ejection fraction, which has improved due to certain factors [36]. They form a heterogeneous group of patients regarding the management, prognosis, and risk of cardiovascular events. Compared to patients with HFpEF, patients with HFrEF are younger, predominantly male, and have a lower prevalence of coronary artery disease, hypertension, DM, chronic lung disease, and atrial fibrillation [37]. However, the identification of these patients can be quite challenging.

## Conclusion and Further Perspective

Studies evaluating the clinical outcomes of patients with HFimpEF have consistently shown a reduction in mortality and hospitalization rate and a better quality of life compared to patients with HFrEF, heart failure with mild reduced ejection fraction, and HFpEF [37, 38, 39, 40, 41, 42, 43]. In patients with heart failure, RR prediction is very important for determining a treatment strategy. We do not yet have an effective model for RR prediction, but it should be comprised of a wide range of information, including historical data and clinical, laboratory, and imaging parameters. However, the development of such a model requires more scrupulous research on this heart failure phenotype. The results of studies focused on RR may, in addition to more accurate RR prediction, also identify new therapeutic targets for patients with HFrEF.

In conclusion, early identification of heart failure, accurate aetiological diagnosis (including genetic testing), and proper treatment with adequate dose titration are key factors for predicting and achieving RR in patients with HFrEF. In-depth study and knowledge of the molecular mechanisms involved in RR may lead to the identification of new individualized therapeutic approaches for HFrEF.

## Conflict of Interest Statement

Dr. Eva Goncalvesova declares the following disclosures (speaker fees, honoraria, consultancy, advisory board fees, investigator, committee member): Servier, AOP Orphan Pharmaceuticals, Boehringer-Ingelheim, Johnson & Johnson, Merck, Novartis, Amgen, Pfizer. Dr. Martin Chudy has no conflicts of interest to declare.

## Funding Sources

The manuscript was supported by a grant from the Slovak Society of Cardiology.

## Author Contributions

Martin Chudý and Eva Goncalvesová were involved in conception, design, literature review, and critical revision of the manuscript and completed, read, and approved the final manuscript.

## Figures and Tables

**Table 1:** Overview of clinical studies aimed at identifying predictors of RR

Study	Studied population	RR criteria	RR observed RR predictors	
Merlo et al. [2], J Am Coll Cardiol 2011	*n* = 242, iDCMP	↑LVEF of ≥10% or ↑LVEF over 50%, ↓LVEDD ≥10% or at ≤33 mm/m^2^	37%	Higher systolic blood pressure, LBBB absence
Amorim et al. [3], Int J Cardiovasc Imaging 2016	*n* = 113, iDCMP	↑LVEF of >10%, ↓LVEDD (not specified), without MR deterioration	34.5%	Mild hypertension, ventricular hypertrophy on ECG, LBBB absence, shorter QRS duration, higher haematocrit, lower LVDDi, higherVO_2_/log 10[VE] and lower dVE/VCO_2_/VO_2_, ACEi/ARB treatment, maximum doses of ACEi/ARB and BB
Matsumura et al. [4], Am J Cardiol 2013	*n* = 19, iDCMP	↓LVEDD to ≤55 mm, fractional shortening improvement to ≥25%	37%	LVRR predictors not evaluated
Kubanek et al. [5], J Am Coll Cardiol 2013	*n* = 44, DCMP (symptoms for less than 6 months)	↑LVEF of ≥10% and to more than 35%, ↓LVEDD of ≥10%	45%	Input predictors: LGE range on CMR and greater myocardial oedema on CMR; after 3 months: BNP value; after 6 months: LVEDDi, E/E' ratio
Hoshikawa et al. [6], Am J Cardiol 2011	*n* = 33, iDCMP	↓LVEDD to ≤55 mm, fractional shortening improvement to ≥25%	42%	No statistically significant differences in the observed predictors
Ikeda et al. [7], Heart Vessels 2015	*n* = 207, iDCMP	↑LVEF of ≥10% and to ≥35%, ↓LVEDDi of ≥10%	52%	LVEDDi decrease during the first 6 months was predictive for LVRR in the later phase
Masci et al. [8], Circ Cardiovasc Imaging 2013	*n* = 58, iDCMP	↑LVEF of ≥10%, ↓LVEDV of ≥10% (according to CMR)	38%	The LGE absence at baseline examination, regardless of the clinical condition and severity of LV dysfunction and dilatation
Luo et al. [9], Chinese J Cardiovac Dis 2021	*n* = 129, HFrEF	↑LVEF of ≥10% and to more than 40%	29.5%	LVEDD ≤55 mm, higher DBP, higher heart rate, the absence of MI
Wilcox et al. [10], Am Heart J 2012	*n* = 3,994, HFrEF or post-IM HFrEF	↑LVEF of ≥10%	28.6%	Female sex, the absence of previous MI, nonischaemic aetiology of HF, no digoxin treatment
Lupón et al. [11], Int J Cardiol 2015	*n* = 304, HFrEF	↑LVEF of ≥15% or ↑LVEF of ≥10% and ↓LVESDi of ≥20% or LVESV of ≥40%	34.2%	ST2-R2 score: ST2 <48 ng/mL, nonischaemic aetiology, the absence of LBBB, HF duration <12 months, baseline LVEF <24%, BB treatment
Choi JO et al. [12], Circ J 2013	*n* = 253, nonischaemic DCMP	(1) ↑LVEF of ≥20% or ≥10% if LVEV reaches ≥50% and (2) ↓LVEDDi of ≥10% or LVEDDi ≤33 mm/m^2^	38%	Higher systolic BP, QRS <120 ms, BB treatment, baseline LVEF, lower LVESDi
Viorel et al. [13], Circ Heart Fail 2016	*n* = 3,519, HFrEF	LVEF >40%	9.1%	Female sex, nonischaemic aetiology, lower BMI, higher DBP, LVEDDi, BB and valsartan treatment
Agra Bermejo et al. [14], Cardiol J 2018	*n* = 449, HFrEF	LVEF >40%	52%	NYHA, ACEi, and BB treatment; nonischaemic aetiology; no ICD implantation
Jung et al. [15], J Cardiovasc Imaging	*n* = 160, DCMP without AF	↑LVEF of >10% or LVEF >50%, ↓LVEDDi of ≥10% or LVEDDi ≤33 mm/m^2^	28%	GLS

ACEi, angiotensin-converting enzyme inhibitors; ARB, angiotensin receptor blockers; BB, beta blockers; BMI, body mass index; CMR, cardiac magnetic resonance; DCMP, dilated cardiomyopathy; DBP, diastolic blood pressure; LVEF, left ventricular ejection fraction; GLS, global longitudinal strain; HFrEF, heart failure with reduced ejection fraction; ICD, implantable cardioverter defibrillator; MI, myocardial infarction; LBBB, left bundle branch block; LGE, late gadolinium enhancement; LVEDD, left ventricular end-diastolic dimension; LVEDDi, left ventricular end-diastolic dimension index; LVESDi, left ventricular end-systolic dimension index; LVRR, left ventricular reverse remodelling; MR, mitral regurgitation.

**Table 2:** The effect of therapy on cellular and molecular changes in LV RR

	(β-Blockers	ACEi	ARB	MRA	LVAD	CRT	ARNI	SGLT2
Cardiomyocyte changes								
Hypertrophy	*↓*	*↓*	*↓*	*↓*	*↓*	*↓*	*↓*	*↓*
Foetal gene expression	*↓*	*↓*	*↓*	ND	*↓*	*↓*	*↓*	
Myocytolysis	*↓*	ND	ND	ND	*↓*	ND	ND	ND
Beta-adrenergic desensitization	*↓*	*↓*	*↓*	ND	*↓*	*↓*	ND	ND
EC coupling	↑	↑	↑	ND	↑	↑	ND	−
Cytoskeletal changes	ND	ND	ND	↑	↑	*↓*	*↓*	ND
Myocardial changes								
Myocyte apoptosis	*↓*	*↓*	*↓*	ND	*↓*	*↓*	*↓*	
MMP activation	*↓*	*↓*	*↓*	*↓*	*↓*	*↓*	*↓*	*↓*
Fibrosis	*↓*	*↓*	*↓*	*↓*	↑	*↓*	*↓*	*↓*
Angiogenesis	↑	↑	↑	↑	*↓*	↑	↑	↑
LV geometry changes								
LV dilatation	*↓*	Stabilization	Stabilization	Stabilization	*↓*	*↓*	*↓*	*↓*

ACEi, angiotensin-converting enzyme inhibitors; ARB, angiotensin receptor blockers; CRT, cardio resynchronization therapy; EC, excitation-contraction; LVAD, left ventricular assist device; MRA, mineralocorticoid receptor antagonists; ND, no data.
